# Upwelling Enhances Mercury Particle Scavenging in the California Current System

**DOI:** 10.1021/acs.est.4c04308

**Published:** 2024-08-22

**Authors:** Xinyun Cui, Hannah M. Adams, Michael R. Stukel, Yiluan Song, Amina T. Schartup, Carl H. Lamborg

**Affiliations:** † Department of Ocean Sciences, University of California Santa Cruz, Santa Cruz, California 95064, United States; ‡ Scripps Institution of Oceanography, University of California San Diego, La Jolla, California 92037, United States; § Department of Earth, Ocean, and Atmospheric Science, Florida State University, Tallahassee, Florida 32306, United States; ∥ Department of Environmental Studies, University of California Santa Cruz, Santa Cruz, California 95064, United States; ⊥ Michigan Institute for Data and AI in Society, University of Michigan, Ann Arbor, Michigan 48109, United States; # Institute for Global Change Biology, University of Michigan, Ann Arbor, Michigan 48109, United States

**Keywords:** Hg cycling, biogeochemistry, California Current

## Abstract

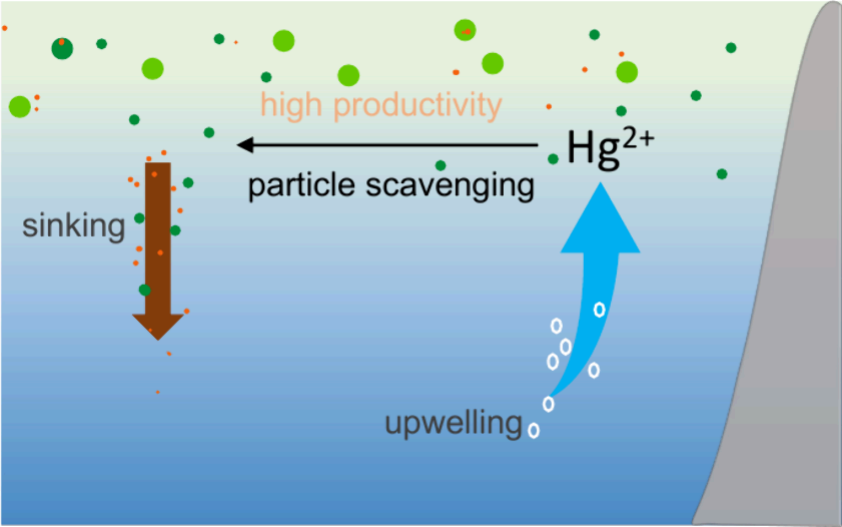

Coastal upwelling supplies nutrients supporting primary production while also adding the toxic trace metal mercury (Hg) to the mixed layer of the ocean. This could be a concern for human and environmental health if it results in the enhanced bioaccumulation of monomethylmercury (MMHg). Here, we explore how upwelling influences Hg cycling in the California Current System (CCS) biome through particle scavenging and sea-air exchange. We collected suspended and sinking particle samples from a coastal upwelled water parcel and an offshore non-upwelled water parcel and observed higher total particulate Hg and sinking flux in the upwelling region compared to open ocean. To further investigate the full dynamics of Hg cycling, we modeled Hg inventories and fluxes in the upper ocean under upwelling and non-upwelling scenarios. The model simulations confirmed and quantified that upwelling enhances sinking fluxes of Hg by 41% through elevated primary production. Such an enhanced sinking flux of Hg is biogeochemically important to understand in upwelling regions, as it increases the delivery of Hg to the deep ocean where net conversion to MMHg may take place.

## Abstract 2

The role of particles is critical to Hg cycling yet is understudied. Leveraging data from upwelling and non-upwelling regions in the California Current Ecosystem, we show that coastal upwelling can enhance Hg sinking flux. This finding implies the potential for increased production of methylated Hg within particles in deeper oceans under upwelling conditions.

## Introduction

Biogeochemical cycling of mercury (Hg) in the ocean is governed by a combination of biotic and abiotic processes that occur within the water column. The distribution of Hg, especially as monomethyl mercury (MMHg), in the ocean has implications for human health, particularly through the consumption of fish.^1^ The California Current System (CCS) is a coastal upwelling biome that supports high primary production and active fisheries.^2,3^ Upwelling brings Hg-enriched water to the mixed layer.^4,5^ However, high primary production^6^ supports substantial export production mediated by sinking particles and hence could potentially return the upwelled Hg to deeper water quickly through scavenging. Moreover, upwelling might also increase elemental Hg (Hg^0^) evasion due to elevated biological reduction of inorganic divalent Hg (Hg^2+^).^7,8^ These enhanced sources and sinks of Hg lead to a complex system with behavior that is difficult to predict. In this study, we present measurements and modeling efforts aimed at unraveling this biogeochemical puzzle.

While many studies have shown that upwelling increases the input of Hg species to the mixed layer,^5,9,10^ there is evidence suggesting that high primary productivity increases particle scavenging within these regions. Figueiredo et al. proposed that upwelling might be contributing to the Hg fluxes on the southern Brazilian continental shelf.^11^ Zaferani et al. also found high Hg export to sediment in the Peruvian upwelling region and elevated Hg accumulation rates in Southern Ocean sediment,^12,13^ suggesting high primary productivity increases particle scavenging and settling of Hg. Therefore, particle scavenging appears to stand out as a mechanism for the removal of Hg from the surface ocean within upwelling regions.

An understudied but critical component of the marine Hg cycle is the role of Hg-bearing sinking particles in Hg transformation and movement. Sinking particles appear to develop anaerobic microenvironments in low-oxygen water, which could support increased anaerobic microbial activities, including biotic Hg methylation that converts Hg^2+^ to MMHg.^14−23^ Furthermore, sinking particles act as a pump that transports Hg species from shallow to deeper waters, thereby reducing the availability of Hg to surface food webs while augmenting its availability to the food web at depth.^24,25^ To date, however, there has been little direct examination of the amount and speciation of Hg associated with sinking particles.

Hg^0^ evasion, another significant sink of Hg in the ocean, could reduce the amount of Hg available for conversion to MMHg. While both biologically mediated and photochemical processes can reduce Hg^2+^ in the water column to Hg^0^, biotic reduction is particularly prevalent in highly productive zones, such as upwelling regions.^7,8^ Nevertheless, Soerensen et al.^26^ indicated that increased ocean productivity may decrease seawater Hg^0^ concentrations through enhanced particle scavenging of Hg^2+^ in the Equatorial Pacific upwelling region, complicating our ability to understand and predict the effect of upwelling on Hg biogeochemistry.

Here, we collected and quantified suspended particulate, sinking particulate, and dissolved Hg species and used these data to quantify the fluxes of Hg species from the surface to intermediate water. Based on our field observed data, we developed a mass balance model to simulate Hg-related inventories and fluxes in the upper 100 m ocean under two scenarios: upwelling and non-upwelling. This modeling approach enables us to describe and quantify several major Hg-related processes and inventories during the upwelling season within the CCS, providing insights into their responses to changes in the upwelling intensity.

## Methods

### Cruise Overview

**Figure 1:**
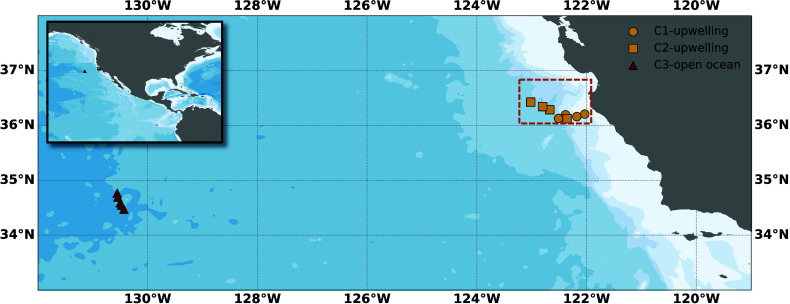
Map of particle sampling stations from the 2021 CCE-LTER Cruise, including upwelling cycle 1 (yellow circle), cycle 2 (yellow square), and open ocean cycle 3 (red triangle). The dashed rectangle represents the modeled domain.

The 2021 California Current Ecosystem Long-Term Ecological Research (CCE-LTER) process cruise (P2107; Stukel and Barbeau, Chief Scientists; Figure 1) focused on tracking coastal upwelling filaments off the central California coast from July to August 2021. We conducted three quasi-Lagrangian experiments (hereafter referred to as “cycles”).^27^ The first two cycles (C1 and C2) followed one upwelled water parcel, and the third cycle (C3) followed a non-upwelled parcel located farther offshore in oligotrophic conditions.

### Particle Sampling and Collection

Suspended particles were collected using McLane Research *in situ* pumps (WTS-LV) using modified, two-layer filter holders.^28^ The top filter holder layer was loaded with a 51 μm polyester mesh screen to collect large-sized fraction particles (LSF). The bottom filter holder layer was loaded with a precombusted GF/F glass microfiber filter (Whatman) to collect small-size fraction particles (SSF, 1–51 μm). Filter holders were acid-leached before use, and the GF/F filters were combusted at 450 °C for 4 h. Sinking particles were collected using VERTEX-style particle-interceptor tube sediment traps.^27^ Sediment traps were deployed during each cycle at depths of 100, 150, and 440 m for 3 or 4 days. Trap tubes were deployed with salt brine (filtered seawater +50 g L^–1^ NaCl). Tubes used for particulate organic carbon (POC) sampling included formaldehyde to prevent microbial activity. After recovery, samples were carefully inspected at 20× magnification, and zooplankton “swimmers” were removed prior to filtration through GF/F filters. For SSF and sediment traps, 2.5 cm diameter filters were stored at −20 °C for further Hg determination. For LSF, 14.2 cm filters were cut in half with ceramic scissors and frozen prior to further analysis.

### Mercury Species and Carbon Determination

Total Hg (THg) and MMHg in suspended and sinking particles were determined by digesting filter portions in nitric acid (2N, trace metal grade, Fisher) for 4 h at a 60 °C constant water bath with intermittent sonication.^10,29^ For THg determination, digestates were oxidized with bromine monochloride for at least 2 h and prereduced with hydroxylamine hydrochloride. Subsamples were then reduced with stannous chloride, and the evolved Hg^0^ concentrations were determined by dual gold amalgamation cold vapor atomic fluorescence spectrometry (CVAFS) with a Tekran 2600 against both gaseous Hg^0^ and aqueous Hg^2+^ standards. MMHg determination used a direct ethylation method.^30^ Digestates were treated with ascorbic acid, buffered with acetate, and neutralized with potassium hydroxide (KOH; 45%) to pH between 4 and 5. Sodium tetraethylborate (1%, in 2% KOH) was added in digestates reacting for 20 min prior to analysis by CVAFS. All reagents above were prepared according to U.S. EPA Method 1630 and 1631.^31,32^ The method detection limit for THg and MMHg were 26.5 and 3.3 fM in the SSF and sinking particles and 0.75 and 0.24 fM in the LSF, respectively. The recovery rates typically ranged from 100 to 110% in our lab. All data are available from BCO–DMO (DOI:10.26008/1912/bco-dmo.926959.1).

Dissolved THg was analyzed following the same procedure as particulate THg described above, excluding filter digestion. Dissolved Hg^0^ was concentrated onto a gold trap by purging seawater samples with Hg-free N_2_. Both THg and Hg^0^ were quantified on board via CVAFS on a Tekran 2600. The sampling depth range is 0 to 200 m. Detailed analytical methods and data are reported in Adams et al.^33^

For total carbon (C) mass determination in the SSF and LSF, filters were dried and packed into tin capsules and analyzed for particulate carbon mass and measured using a Carlo Erba 1108 elemental analyzer coupled to a Thermo Finnigan Delta Plus XP IRMS at the University of California, Santa Cruz. The analytical precision of C % is 0.65% in the SSF and 1.1% in the LSF based on the organic standards IU Acetanilide and USGC41. The recovery rate is 100.87 ± 0.87% (*n* = 4). Here, we use total C mass as a proxy for POC, since it comprises a significant portion (>90%) of total carbon in the suspended particles.^34,35^

### Sinking Flux Calculation

Field-observed Hg and C sinking fluxes (pmol Hg m^–2^ d^–1^ or μmol C m^–2^ d^–1^) are estimated as follows:
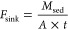
1where *M*_sed_ is the amount of Hg species (pmol Hg) or organic carbon (μmol C) from the sediment trap and *A* and *t* represent the sediment trap area (m^2^) and deployment duration (d).

### Hg Partitioning Coefficient (*K*_OC_) Calculation

Here, we use the partitioning coefficient (*K*_OC_), defined as the apparent affinity of Hg for marine POC. We use particulate and dissolved Hg data to calculate *K*_OC_ (unit: L kg^–1^) for the SSF and LSF particles, following the expression as
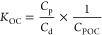
2where *C*_p_ and *C*_d_ represent the concentration (pM) of Hg in particulate and dissolved phases, respectively. *C*_POC_ represents the concentration (kg L^–1^) of POC. We use carbon mass as a proxy for suspended solids, as the typical partitioning coefficient calculation (*K*_d_)^36,37^ requires suspended particulate mass data, which is unavailable in our study. As organic carbon makes up approximately 20–50% of the total particulate mass in the upper 500 m of a continental shelf region,^28,38,39^ an estimate of *K*_d_ (if one wanted to compare to other values in the literature) can be made from our *K*_OC_ values by dividing by 2–5.

### General Model Description

Our model is modified from Liu et al.^40^ by explicitly accounting for upwelling-related fluxes and is applied to the coastal upwelling region of the CCS with latitude ranging from 36°N to 36.7°N and longitude from 121.5°W to 123.2°W (about 188 km in cross-shore distance, 77 km in along-shore distance, and 100 m in depth; Figure 1).

We applied a Monte Carlo approach comprised of 1000 trials (Figure S2) to generate the distributions of key inventories and processes, including dissolved Hg^2+^, Hg^0^, particulate Hg^2+^, evasion, atmospheric deposition, redox reactions, particle scavenging, water exchange, and upwelling input with averaged upwelling velocity determined by the Coastal Upwelling Transport Index (CUTI).^41^ Upwelling input was modeled as the product of the upwelling velocity and Hg concentration below domain depth. We incorporated upwelling input into calculations of evasion, atmospheric deposition, redox reactions, particle sinking, and water exchange to determine the rate of change in Hg^0^ and Hg^2+^ concentrations. Detailed descriptions and equations are provided in eqs S1–S5 Riverine input was omitted from our model, as only two small rivers flow into our study domain. Likewise, the influence of atmospheric dust deposition on marine particles was ignored due to the dominance of biogenic particles in this region^39^ (see detailed discussion in the SI). Each trial begins with a random selection of parameter values from their respective distributions. The simulation covered 60 days, using an hourly timestep until all monitored inventories and fluxes reached a steady state.

Some parameters were specifically tailored to the CCS based on literature and field observations (Table S1), while others were adapted from Liu et al.’s model.^40^ Additionally, we performed a comparative analysis with a nonparametric test by repeating the procedure with a modified scenario where the upwelling velocity was 0 m d^–1^. In this scenario, net primary productivity (NPP), export, and POC values were obtained from the open ocean (Table S1). The model was evaluated by comparing its output to the field observations obtained during our cruise.

To better constrain modeled Hg inventories and fluxes, we used a sensitivity analysis to assess the stability of model output and robustness of findings in the case of possible bias in parameter estimation. We focused on wind speed and *K*_OC_ due to their importance in estimating the sinking flux and evasion. We evaluated the changes in model output by changing one parameter at a time while keeping all other parameters unaltered. The selected values were chosen from the 10th, 50th, and 90th percentiles of their respective distributions.

## Results & Discussion

### 1 Elevated Suspended Particulate Hg Species Concentrations and Lower *K*_OC_ in the Upwelling Region Compared to the Open Ocean

**Figure 2:**
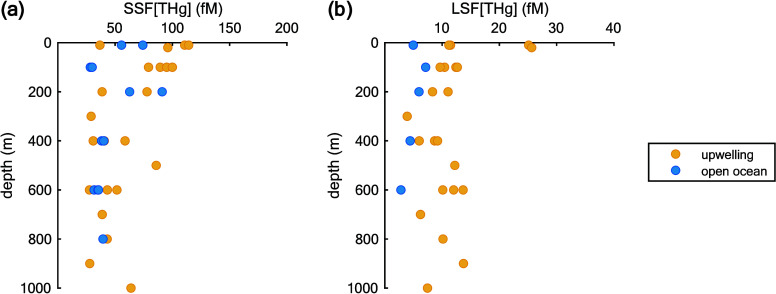
Profiles of THg concentration (fM) distribution with depth (m) for the upwelling (yellow) and open ocean (blue) regions. Panel (a) is for THg in the SSF, while panel (b) is for THg in the LSF.

Profiles of particulate THg for SSF and LSF are listed in Figure 2. The concentrations vary with depth, but no statistically significant trend is observed (*p*-value > 0.05). The THg concentration is much higher in the SSF (28 to 114 fM) and has a wider concentration range than the LSF (2 to 25 fM) across all stations (Table 1, *p*-value < 0.05) due to higher concentrations of SSF in the water column.^28^ The upwelling regions exhibited higher particulate THg than the open ocean (*p*-value < 0.05). In the upper ocean (above 200 m), the ratio of particulate THg to dissolved THg averaged 14.55% (range: 3–47%), which is higher than the ratios reported in the Equatorial Pacific,^10^ indicating that particles are an important phase of Hg in the coastal upper ocean.

**Table 1:** Range, Mean ± Standard Deviation of THg and MMHg Concentrations in SSF and LSF for Upwelling and Open Ocean, Respectively^a^

		SSF[THg] (fM)		LSF[THg] (fM)		SSF[MMHg] (fM)		LSF[MMHg] (fM)	
regions	depth (m)	range	mean ± std	range	mean ± std	range	mean ± std	range	mean ± std
upwelling	0–100	37–114	90.1 ± 24.3	9–25	14.8 ± 6.6	2–19	8.6 ± 7.7	0.4–1.8	2.3 ± 2.7
	>100	27–86	47.0 ± 18.5	4–14	9.5 ± 3.0	1– 10	6.9 ± 3.5	0.1–2.1	0.8 ± 0.6
open ocean	0–100	30–74	47.0 ± 22.0	5–7	6.0 ± 1.5	NA	NA	0.1–0.2	0.2 ± 0.04
	>100	31–91	46.7 ± 20.3	3–6	4.4 ± 1.6	12	NA	2.8	NA

a “NA” indicates that there is no available data.

**Figure 3:**
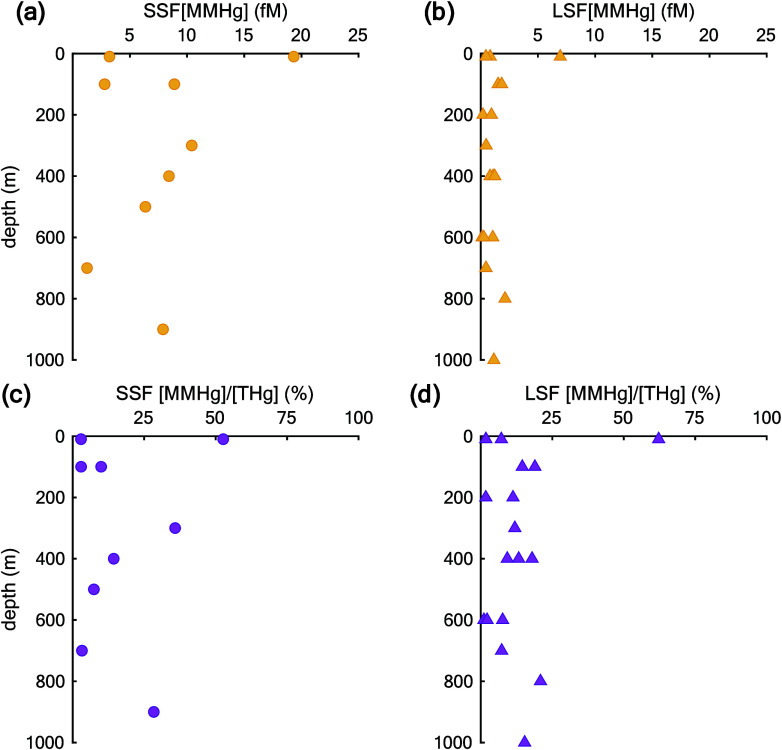
Profiles of MMHg concentration (fM) and MMHg/THg ratio (%) distribution with depth (m) for the upwelling regions. Panels (a) and (b) in yellow are for MMHg concentrations in the SSF and LS; panels (c) and (d) in purple are for MMHg/THg ratios in the SSF and LSF, respectively. Circles represent SSF, while triangles represent LSF.

For MMHg, we only analyzed particulate data from stations in the upwelling region, with a range of 2–19 fM in the SSF and 0.1–2 fM in the LSF (Table 1, Figure 3a,b). We observed a significantly higher average MMHg to THg ratio in the SSF at 19.17% compared to the LSF at 11.69% (*p*-value < 0.05; Figure 3c,d), suggesting that MMHg production may be favored on SSF particles within the CCS. As noted above, particles may provide an anaerobic environment for Hg methylation; however, there are few studies on the preference of MMHg for particles across the size spectrum.^16,23^ While it has been reported that LSF can sustain an anaerobic environment for sulfate-reducing bacteria even in an oxygenated water column,^16^ our finding suggests that the SSF may serve as an equal or more favorable microenvironment for MMHg production or retention. This is consistent with Hg isotope data that has suggested that microbial methylation of Hg is more likely to occur on SSF than LSF in the Pacific Ocean.^23^

Interestingly, MMHg/THg ratios in sediment traps averaged 2.15% (Table S2), a much lower ratio than that of SSF and LSF. This discrepancy in ratio may be driven by zooplankton grazing, as zooplankton preferentially bioaccumulate MMHg and excrete inorganic Hg^2+^;^42^ thus, the fecal particles they produce^43,44^ are depleted in MMHg relative to ingested prey,^23,45^ which are mostly small suspended particles.^45,46^ Because zooplankton fecal pellets typically dominate the sinking flux in the CCS (usually >50% and often >90% during highest flux conditions),^47,48^ this mechanism could explain the decrease in MMHg/THg ratios in sinking particles compared to suspended particles.

The log *K*_OC_ of THg in the SSF is 7.2 ± 0.3 in the open ocean and 6.6 ± 0.5 in the upwelling region. This is in line with the previous finding of Cui et al.^37^ that the open ocean generally exhibits higher *K*_d_ values when compared to the coastal regions (*p*-value < 0.05) due to the so-called particle concentration effect where *K*_d_ displays an inverse relationship with particle mass.^49,50^ Notably, the upwelling region shows a higher POC concentration (*p*-value < 0.05), averaging 28.1 μg/L, compared to the open ocean (7.6 μg/L), showing a concentration gradient that is consistent with the particle concentration effect on *K*_OC_ values (Figure S1). Additionally, the value of *K*_OC_ in the SSF is comparable to that in the LSF in both regions (LSF log *K*_OC_, upwelling: 6.6 ± 0.4; open ocean: 7.42 ± 0.1) (*t* test; *P*-value > 0.05). This similarity indicates that the SSF and LSF have similar adsorption properties. Further investigations are necessary to explore the adsorption/desorption kinetics of Hg onto POC, including the characterization of chemical groups within POC and the quantification of binding sites available on POC, to improve understanding of the Hg scavenging process associated with particle composition.

Compared to THg, the mean log *K*_OC_ for MMHg in the upwelling region was 5.7 ± 0.6, indicating that MMHg is less strongly scavenged onto particles compared to inorganic Hg (demonstrated by the *K*_OC_ for THg). This is consistent with previous findings in both fresh and saltwater, where the *K*_d_ for MMHg is generally reported to be 10 times lower than that for THg.^51^

### 2 CCS Upwelling Increases Hg Sinking Flux and Evasion

Sinking fluxes for THg, MMHg, and POC are summarized in Table 2. POC attenuates with depth as a result of substantial remineralization and biological consumption.^52^ Given the consistently strong affinity of Hg for POC, we would anticipate a similar decreasing trend of Hg sinking fluxes with depth. However, THg sinking fluxes did not attenuate with depth in the upwelling region; they exhibited a maximum at 150 m. This implies that Hg might continue to be scavenged onto particles as they sink. We propose that the loss of POC allows the remaining components in particles to exert greater control on the adsorption to Hg. For example, while the apparent affinity of iron (Fe) oxyhydroxides for Hg is the strongest of any phase, their contribution to overall Hg *K*_d_ is relatively small due to their small contribution to overall particulate mass in shallow water. As the POC contribution to sinking particle mass decreases due to remineralization, however, Fe oxides should be expected to become more influential in adsorbing Hg onto particles and raise the overall *K*_OC_.^37^ There is preliminary evidence of subsurface particulate Fe maxima from the immediately previous cruise that aligns with our hypothesis (Allison Laubach, personal communication). Additionally, subsurface peaks of particulate Fe have been observed in other continental margins due to lateral transport (e.g., subarctic Pacific).^53,54^ Therefore, Fe might be a key mechanism driving the observed subsurface maxima of Hg in the upwelling region.

**Table 2:** Sinking Fluxes of THg, MMHg, and C Mass in the Upwelling (Cycles 1 and 2) and Open Ocean (Cycle 3) Regions at Different Depths^a^

		sinking flux		
regions	depth (m)	THg (pmole m^–2^ d^–1^)	MMHg (pmole m^–2^ d^–1^)	POC (μmol m^–2^ d^–1^)
upwelling (C1)	100	1517	23	16750
	150	2879	NA	13333
upwelling (C2)	100	720	10	8667
	150	1370	46	7750
open ocean (C3)	150	1479	6	3583
	440	772	32	2583

a “NA” indicates that there are no available data.

The high sinking fluxes of Hg species observed in the CCS are 1 order of magnitude higher than those reported in the Equatorial Pacific.^10^ This is consistent with higher productivity in the CCS compared with the Equatorial Pacific. Upwelled deep waters are a major source of nutrients to support primary production and stimulate particle formation and settling.^55^ In the CCS, primary production was reported to have a significant positive relationship with C export.^56^ We observed higher NPP and export in the upwelling region compared to the open ocean during our cruise (Table S1). Thus, we would anticipate the high Hg export within CCS upwelling region due to the high affinity of Hg to POC.^37^ Similar observations of high Hg sedimentation related to sorption and scavenging have been reported in other upwelling sites, such as the Antarctic and Peruvian upwelling regions, with values at the same order of magnitude as ours.^11−13^

**Figure 4:**
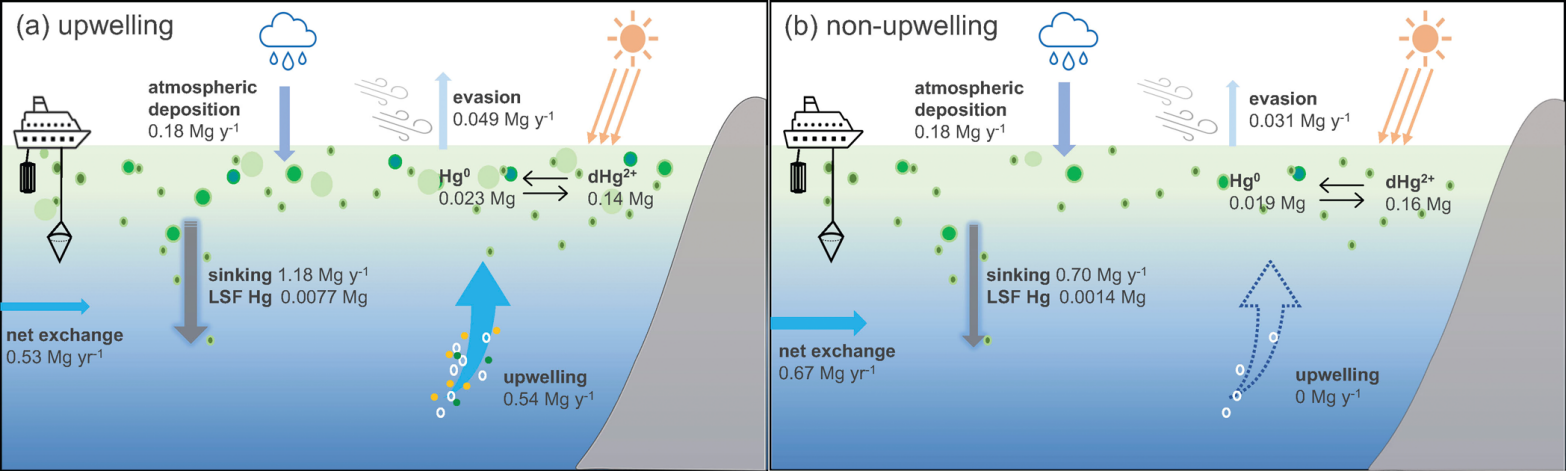
Simulated Hg budgets in the coastal CCE domain under two scenarios: (a) upwelling with vertical velocity of 1.0 m d^–1^ and (b) non-upwelling.

To test the response of Hg-related processes and species inventories due to upwelling, we developed a box model for our study region in the CCS. Modeled Hg budgets in the upper ocean within the CCS upwelling region are presented in Figure 4a and Table S3. Table 3 shows the agreement between the simulated and field-observed values. Simulation results demonstrate that the Hg sinking flux is the predominate removal pathway for Hg, accounting for 96% of Hg sinks from the surface ocean, exceeding the loss from evasion. On the other hand, upwelling supplies 42% of dissolved and particulate Hg species to the surface ocean. Hence, the sinking flux surpasses the upwelling input and drives Hg removal from the surface.

**Table 3:** Model Simulation Data (Median) and Field Observation Data (Mean) for Sinking Flux, Particulate THg, Dissolved THg, and Elemental Hg Across a Depth Range of 0–100 m^a^

variable	unit	modeled (median)	measured (mean)
sinking flux	pmole m^–2^ d^–1^	1115 (440, 2267)	1118 (720, 1517)
LSF THg	fM	26.6 (2.8, 78.4)	14.8 (9, 25)
dissolved THg	pM	0.6 (0.3, 0.9)	0.6 (0.2, 1.2)
dissolved Hg^0^	fM	78 (39, 156)	67 (19, 160)

a Modeled values in parentheses represent the 90% prediction interval, and measured values in parentheses represent the range.

To better understand the effect of upwelling on Hg sinking flux, we ran the model with an upwelling speed of 1.0 m d^–1^, (eq S3, Table S1), and no upwelling. In the absence of upwelling, the model predicts a 41% decreased sinking flux (Figure 4b) compared to the 1.0 m d^–1^ upwelling velocity scenario, indicating that the Hg sinking flux has a positive relationship with upwelling. Upwelling promotes NPP resulting in increased particle production, which, in turn, enhances Hg scavenging and subsequent removal. Thus, without upwelling, there is reduced particle production and less scavenging activity. Furthermore, the comparison of results from the two scenarios reveals that upwelling not only influences Hg sinking flux but also plays a significant role in other Hg processes, such as Hg evasion. Our simulations indicate a 45% increase in Hg evasion under upwelling conditions compared to non-upwelling conditions, consistent with previous observations within CCS that Hg evasion is facilitated by upwelling-induced cyclonic eddies.^5^ This is partially due to upwelling supplying additional Hg^0^ to the surface ocean, increasing the concentration gradient between the surface ocean and the atmosphere that drives Hg^0^ evasion. Additionally, the prevalent biotic reduction rate in highly productive regions increases, increasing Hg^0^ concentrations through the reduction of Hg^2+^, which is also brought to the surface from upwelling.^57^ The intensified sinking and Hg^0^ evasion effectively remove substantial amounts of Hg from the mixed layer, ultimately resulting in a lower concentration of THg in the mixed layer.

**Figure 5:**
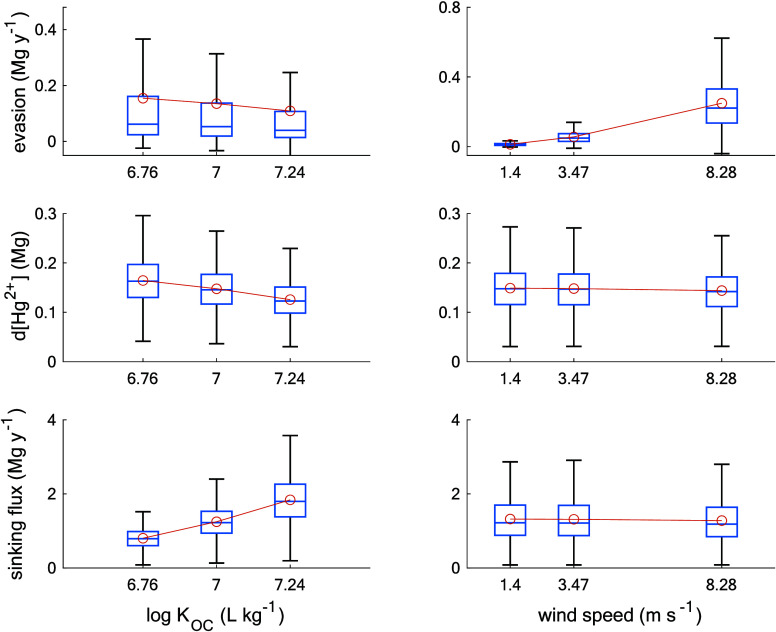
Sensitivity analysis of evasion, dissolved Hg^2+^, and sinking flux, to log *K*_OC_ and wind speed parameters. Values of log *K*_OC_ and wind speed were selected from the 10th, 50th, and 90th percentiles of their distribution. Box plots display the median, the lower and upper quartiles, the whiskers connecting quartile to nonoutlier maximum/minimum, and the mean represented by orange circle.

We tested the sensitivity of model results to a group of parameters that we suspected were the most influential, including *K*_OC_ and wind speed (Figure 5). *K*_OC_ impacts various Hg processes including sinking flux, evasion, and dissolved Hg^2+^ inventory. For instance, across the 10th, 50th, and 90th percentile of log *K*_OC_, the sinking flux increased by 128%, while evasion decreased by 35%, and the dissolved Hg^2+^ pool decreased by 25%. This is consistent with the idea of competition between particle scavenging and reduction/evasion, as both processes remove Hg^2+^ from the dissolved pool, but competition is not always apparent as productivity may influence the reduction rate as well. Conversely, wind speed demonstrates a relatively minor effect on the overall Hg cycle as it primarily influences evasion. Specifically, across the 10th, 50th, and 90th percentile of wind speed, evasion increased by 19 times, while the dissolved Hg^2+^ pool and sinking flux exhibited a subtle response, with a decrease of 4% each. It is worth noting that while the various parameters have a crucial role in determining the magnitude of Hg processes, they do not alter the fundamental premise that upwelling enhances Hg sinking flux.

### 3 Possible Implications of Intensified Coastal Upwelling

Coastal upwelling is expected to intensify as a consequence of global warming.^6,58^ Our model suggests a decrease in the amount of bioavailable dissolved Hg^2+^ in the upper ocean, primarily driven by enhanced particle scavenging and Hg^0^ evasion. This reduction in Hg concentration in the mixed layer implies a lower availability of Hg to epipelagic marine organisms, thereby mitigating the potential threat to fish and humans. However, the increased downward export of Hg and C caused by upwelling probably would result in more in situ production of total methylated Hg (MeHg; sum of MMHg and dimethylmercury) in deeper water under lower oxygen conditions, followed by MMHg release to seawater.^59^ Elevated MeHg concentration at depth, observed during a previous cruise in the CCS,^5^ indicates that the mesopelagic ocean is an important source of MeHg. Therefore, it is crucial to explore the role of the mesopelagic source of MeHg in contributing to surface MeHg levels through upward transport. This will enhance our understanding of how upwelling influences Hg dynamics and its subsequent incorporation into the surface food web.

Overall, intensified upwelling can remove substantial Hg from the surface to deep water as shown by field observation and simulation, likely reducing the impact of Hg on the surface food web and fish, but this effect might be complicated by the upward transport of MeHg generated in the mesopelagic ocean, influencing the food web in the surface ocean.

## Supporting Information Available

Model detailed description (eqs S1–S5, Table S1); field-observed data analysis (Table S2, Figure S1); model outputs (Table S3, Figure S2) (PDF)
