# Supplementary material for: Upwelling Enhances Mercury Particle Scavenging in the California Current System
Source: Environ Sci Technol. 2024 Aug 22;58(35):15598–606. doi: 10.1021/acs.est.4c04308 (PMC11375766; doi:10.1021/acs.est.4c04308)
Supplement: Supplementary file 1 — es4c04308_si_001.pdf [file es4c04308_si_001.pdf]

Supporting Information

Upwelling Enhances Mercury Particle Scavenging in the California Current System

Xinyun Cui[1*], Hannah M. Adams[2], Michael R. Stukel[3], Yiluan Song[4], Amina T. Schartup[2], Carl H. Lamborg[1]

[1]Department of Ocean Sciences, University of California Santa Cruz, Santa Cruz, CA 95064, USA

[2]Scripps Institution of Oceanography, University of California San Diego, La Jolla, CA 92037, USA

[3]Department of Earth, Ocean, and Atmospheric Science, Florida State University, Tallahassee, FL 32306, USA

[4]Department of Environmental Studies, University of California Santa Cruz, Santa Cruz, CA 95064, USA

Corresponding Author: Xinyun Cui, xcui12@ucsc.edu

Summary: 7 pages, 3 tables, 2 figures, and 5 equations

Table of Contents

Model Description 2
Field Observed Data Analysis 5
Model Outputs 6
Reference 8

Model Description

Model Variable

Air-sea exchange of elemental Hg (F_{eva}; ng L^{-1} h^{-1}) is modeled as in in Soerensen et al.[1] ; F_{eva} is set equal to:

$$F_{eva} = k_{eva}\left([Hg^0]_{sea} \times 1000 - \frac{[Hg^0]_{atm}}{H(S,T)}\right)$$

[equation S1]

Where $[Hg^0]_{sea}$ is the Hg^0 concentration in surface seawater (pM), $[Hg^0]_{atm}$ is the Hg^0 concentration in the air (also in pmole/L units), H is the unitless Henry's Law constant and a function of temperature and salinity (Andersson et al., 2008)[2] and k_{eva} is the mass transfer coefficient (also called a "piston velocity"), calculated using the Nightingale et al. formulation.[3]

Atmospheric deposition of Hg^{2+} (F_{dep}; ng L^{-1} h^{-1}) is estimated using the GEOS-Chem atmospheric chemical transport model (version 13.1.2, DOI: https://doi.org/10.5281/zenodo.5075847).

The sinking flux of Hg (F_{sink}: ng L^{-1} h^{-1}) is modeled by multiplying particulate Hg by a particle deposition rate. The particle deposition rate (v_{dep}: m^2 d^{-1}) is calculated as export (mgC m^{-2} d^{-1}) divide by particulate organic carbon (POC; mgC m^{-3}) (Table S1).

$$F_{sink} = [Hg]_p \times v_{dep} \times \frac{1\,d}{24\,h}$$

$$V_{dep} = \frac{export}{POC}$$

[equation S2]

The upwelling flux of Hg ($F_{upwelling}$; ng L^{-1} h^{-1}) is estimated by multiplying the vertical upwelling velocity of water by THg concentration in the intermediate seawater just below the

model depth boundary. Upwelling velocity is estimated by dividing upwelling index (UI; m^2 d^{-1}) by upwelling cross-shore length (UL; m) (Table S1).

$$F_{upwelling} = [Hg] \times v_{upwelling} \times \frac{1\,d}{24\,h}$$

$$v_{upwelling} = \frac{UI}{UL}$$

[equation S3]

Hg export to and import from the neighboring ocean (F_{ex}, unit: ng L^{-1} h^{-1}) is simplified and expressed as water mass exchange rate multiplied by the THg concentrations in the "upstream" water mass (flux in), or the THg concentration within the model domain (flux out). We follow the method of Liu et al. to simplify the water mass exchange rate by dividing domain water volume (V) by water residence time (rt, unit: d).[4]

$$F_{ex} = \frac{[Hg] * V}{rt} \times \frac{1\,d}{24\,h}$$

[equation S4]

The total Hg^{2+} and Hg^0 concentration (ng L^{-1}) in the seawater were calculated by integrating the rate of change in fluxes over time (h). Particulate Hg^{2+} (ng L^{-1}) was calculated using an equation based on the partitioning coefficient (K_d) equation [5, 6]. $k_{\text{red-Hg(II)}}$ and $k_{\text{ox-Hg0}}$ are the pseudo-first order reduction rate constant of Hg(II) and oxidation rate (h^{-1}).[4]

$$\frac{\partial[Hg^0]}{\partial t} = -F_{eva} + F_{upwelling} + k_{red-Hg(II)} \times [Hg^{2+}] - k_{ox-Hg0} \times [Hg^0] + F_{ex}$$

$$\frac{\partial[tHg^{2+}]}{\partial t} = F_{dep} - F_{sink} + F_{upwelling} - k_{red-Hg(II)} \times [Hg^{2+}] + k_{ox-Hg0} \times [Hg^0] + F_{ex}$$

$$[Hg]_p = \frac{[POC] \times K_{OC} \times [tHg^{2+}]}{1 + [POC] \times K_{OC}}$$

[equation S5]

Estimate of Atmospheric Dust Deposition

We estimated the Hg flux from atmospheric dust to the ocean, using Hg/Al ratio ($0.5 * 10^{-6}$ g/g) in the south Pacific[7], dust flux (18.4 mg/m^2/d) and Al concentration ($14.1 * 10^2$ μmol /g) in dust in the southern CCS[8]. The estimated Hg flux from dust is 350 pg/m^2/d. The particulate Hg in dust is ~19 ng/g. Although we do not have total particle mass, if we assume that total carbon mass approximately accounts for 20%, then the rough Hg concentration in ng/g, calculated from Hg/C in our study, is 290 ng/g. Therefore, the Hg from dust would only account for 6.5% of total particulate Hg. This suggests that dust is not a big source to particulate Hg in our study region.

Table S1 CCS Parameters

Parameter	Units	Distribution	Source
width	m	77000	
length	m	188000	
depth	m	100	
surface area	m^2	1.45×10^9	
water volume	m^3	1.19×10^{11}	
upwelling cross-shore length (UL)	m	75000	Zaba (2021)[9]
surface water temperature	°C	normal (13.15, 1.18)	P2107
wind velocity	m s^{-1}	lognormal (1.27, 0.72)	CeNCOOS[a]
Shortwave radiation flux	W m-2	normal (255, 35)	CeNCOOS[a]
upwelling index (UI)	m^2 s^{-1}	gamma (0.97, 0.82)	CUTI [10, b]
water residence time	d	uniform (8, 18)	Liu (2019)[11]
NPP	mgC m^{-2} d^{-1}	upwelling: normal (1083, 403)<br>non-upwelling: normal (274, 57)	P2107
export	mgC m^{-2} d^{-1}	upwelling: normal (235, 49)<br>non-upwelling: 125	P2107
POC	mg m^{-3}	upwelling: normal (9.53, 12.08)<br>non-upwelling: normal (0.98, 0.8)	P2107
DOC	mg L^{-1}	normal (0.7, 0.1)	P2107
chlorophyll	mg m^{-3}	lognormal (-0.81, 1.48)	P2107

atmospheric Hg^0 deposition	ng L^{-1} h^{-1}	uniform (1.88, 3.49)×10^{-6}	GEOS-Chem
atmospheric Hg^{2+} deposition	ng L^{-1} h^{-1}	uniform (0.8 ,1.48)×10^{-5}	GEOS-Chem
atmospheric Hg^0 concentration	ng m^{-3}	1.41	Wesis-Penzias (2013)[12]
Hg^{2+} in neighbor ocean	ng L^{-1}	normal (0.13, 0.04)	P2107
Hg^0 in neighbor ocean	pmole L^{-1}	normal (0.015, 0.0082)	P2107
Hg^{2+} in bottom ocean	ng L^{-1}	normal (0.144, 0.027)	P2107
Hg^0 in bottom ocean	ng L^{-1}	normal (0.017, 0.008)	P2107
Hg log K_{OC}	L kg^{-1}	normal (7, 0.18)	P2107

[a] CeNCOOS: https://www.cencoos.org/

[b] CUTI: https://mjacox.com/upwelling-indices/

Field Observed Data Analysis

Table S2 The summary of THg/C, MMHg/C, and MMHg/THg ratios in sinking particles.

regions	depth(m)	Hg/C (pmol μmol^{-1})	MMHg/C (pmol mmol^{-1})	MMHg/THg (%)
upwelling(Cycle 1)	100	0.09	1.37	1.48
	150	0.22	NA	NA
upwelling(Cycle 2)	100	0.08	1.15	1.42
	150	0.18	5.94	3.36
open ocean(Cycle 3)	150	0.41	1.67	0.40
	440	0.30	12.39	4.11

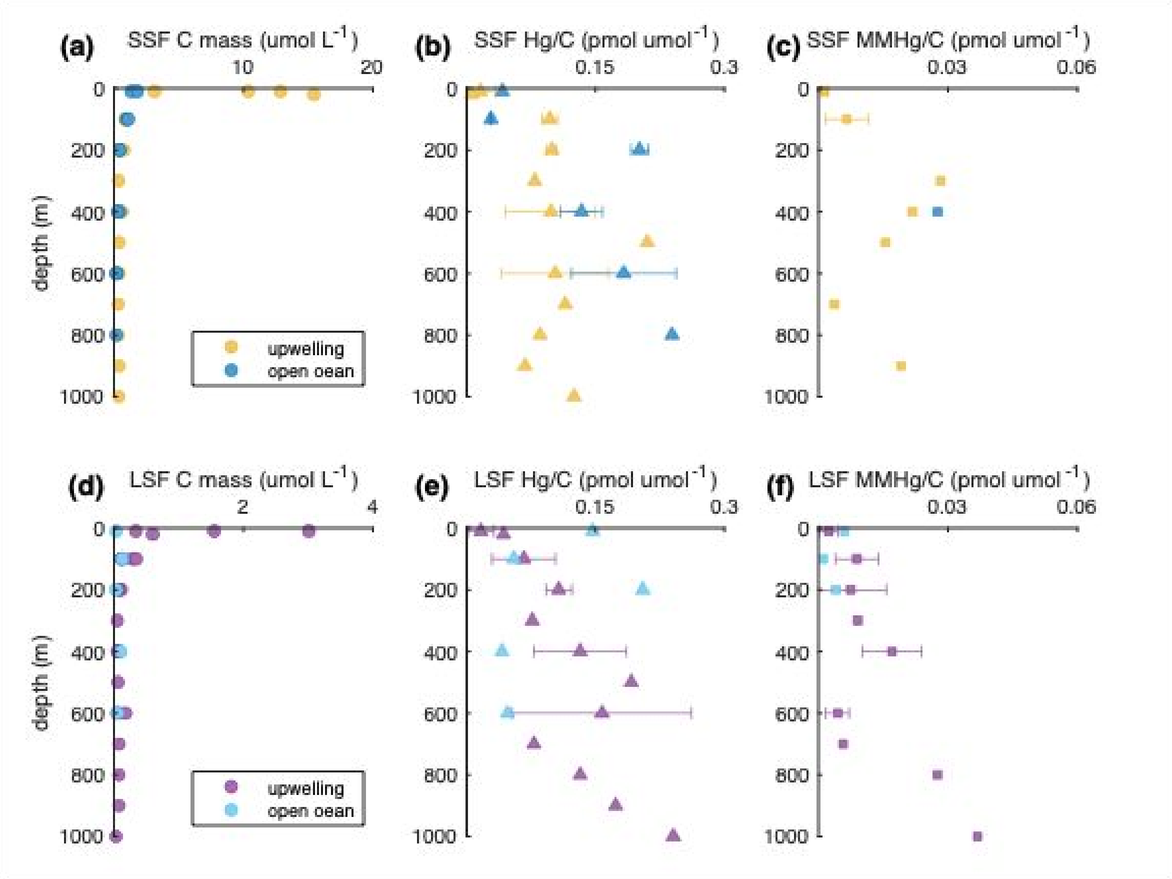

Figure S1

Profiles of carbon (C) mass (μmol L^{-1}), THg to C ratio, and MMHg to C ratio (pmol μmol $^{-1}$) distribution with depth (m) in upwelling and open ocean. Panel (a), (b), and (c) are for the C mass, THg/C ratio, and MMHg/C ratio in SSF, respectively; panel (d), (e), and (f) are for the C mass, THg/C ratio, and MMHg/C in LSF. Error bars represent standard deviations.

Model Outputs

Table S3 Simulated Hg budgets showing median values for upwelling and non-upwelling scenarios. A '0' in non-parametric test indicates no significant difference between two scenarios, while a "1" indicates significant difference.

	unit	upwelling (1.0 m d^{-1})	non-upwelling	non-parametric test
atmospheric deposition	Mg yr^{-1}	0.1792	0.1785	0
water exchange	Mg yr^{-1}	0.5349	0.6668	1
Hg evasion	Mg yr^{-1}	0.0494	0.0310	1
dissolved Hg^{2+}	Mg	0.1427	0.1581	1
particulate Hg^{2+}	Mg	0.0077	0.0014	1
Hg^0	Mg	0.0227	0.0185	1
upwelling input	Mg yr^{-1}	0.5412	0	1
sinking flux	Mg yr^{-1}	1.1780	0.6998	1

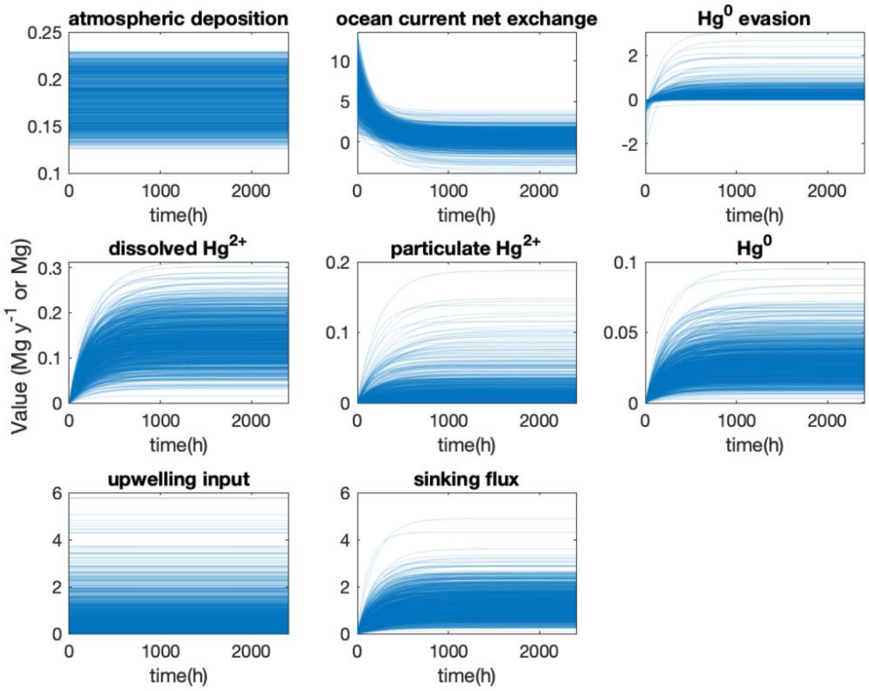

Figure S2 Monte Carlo simulation plots for selected variables.

Reference

(1) Soerensen, A. L.; Sunderland, E. M.; Holmes, C. D.; Jacob, D. J.; Yantosca, R. M.; Skov, H.; Christensen, J. H.; Strode, S. A.; Mason, R. P. An improved global model for air-sea exchange of mercury: High concentrations over the North Atlantic. *Environmental science & technology* **2010**, *44* (22), 8574-8580.

(2) Andersson, M. E.; Gårdfeldt, K.; Wängberg, I.; Strömberg, D. Determination of Henry's law constant for elemental mercury. *Chemosphere* **2008**, *73* (4), 587-592.

(3) Nightingale, P. D.; Malin, G.; Law, C. S.; Watson, A. J.; Liss, P. S.; Liddicoat, M. I.; Boutin, J.; Upstill-Goddard, R. C. In situ evaluation of air-sea gas exchange parameterizations using novel conservative and volatile tracers. *Global Biogeochemical Cycles* **2000**, *14* (1), 373-387.

(4) Liu, M.; Zhang, Q.; Maavara, T.; Liu, S.; Wang, X.; Raymond, P. A. Rivers as the largest source of mercury to coastal oceans worldwide. *Nature Geoscience* **2021**, *14* (9), 672-677. DOI: 10.1038/s41561-021-00793-2.

(5) Bacon, M.; Spencer, D.; Brewer, P. 210Pb/226Ra and 210Po/210Pb disequilibria in seawater and suspended particulate matter. *Earth and Planetary Science Letters* **1976**, *32* (2), 277-296.

(6) Cui, X.; Lamborg, C. H.; Hammerschmidt, C. R.; Xiang, Y.; Lam, P. J. The Effect of Particle Composition and Concentration on the Partitioning Coefficient for Mercury in Three Ocean Basins. *Frontiers in Environmental Chemistry* **2021**, *2*. DOI: 10.3389/fenvc.2021.660267.

(7) Mason, R. P.; Hammerschmidt, C. R.; Lamborg, C. H.; Bowman, K. L.; Swarr, G. J.; Shelley, R. U. The air-sea exchange of mercury in the low latitude Pacific and Atlantic Oceans. *Deep Sea Research Part I: Oceanographic Research Papers* **2017**, *122*, 17-28.

(8) Félix-Bermúdez, A.; Delgadillo-Hinojosa, F.; Huerta-Diaz, M. A.; Camacho-Ibar, V.; Torres-Delgado, E. V. Atmospheric Inputs of Iron and Manganese to Coastal Waters of the Southern California Current System: Seasonality, Santa Ana Winds, and Biogeochemical Implications. *Journal of Geophysical Research: Oceans* **2017**, *122* (11), 9230-9254. DOI: 10.1002/2017jc013224.

(9) Zaba, K. D.; Franks, P. J. S.; Ohman, M. D. The California Undercurrent as a Source of Upwelled Waters in a Coastal Filament. *Journal of Geophysical Research: Oceans* **2021**, *126* (2). DOI: 10.1029/2020jc016602.

(10) Jacox, M. G.; Edwards, C. A.; Hazen, E. L.; Bograd, S. J. Coastal Upwelling Revisited: Ekman, Bakun, and Improved Upwelling Indices for the U.S. West Coast. *Journal of Geophysical Research: Oceans* **2018**, *123* (10), 7332-7350. DOI: 10.1029/2018jc014187.

(11) Liu, X.; Dunne, J. P.; Stock, C. A.; Harrison, M. J.; Adcroft, A.; Resplandy, L. Simulating Water Residence Time in the Coastal Ocean: A Global Perspective. *Geophysical Research Letters* **2019**, *46* (23), 13910-13919. DOI: 10.1029/2019gl085097.

(12) Weiss-Penzias, P. S.; Williams, E. J.; Lerner, B. M.; Bates, T. S.; Gaston, C.; Prather, K.; Vlasenko, A.; Li, S. M. Shipboard measurements of gaseous elemental mercury along the coast of Central and Southern California. *Journal of Geophysical Research: Atmospheres* **2013**, *118* (1), 208-219. DOI: 10.1029/2012jd018463.
